# Accumulation of tissue factor in endothelial cells promotes cellular apoptosis through over-activation of Src1 and involves β1-integrin signalling

**DOI:** 10.1007/s10495-019-01576-2

**Published:** 2019-10-25

**Authors:** Ali M. Ethaeb, Mohammad A. Mohammad, Yahya Madkhali, Sophie Featherby, Anthony Maraveyas, John Greenman, Camille Ettelaie

**Affiliations:** 1 Biomedical Sciences, University of Hull, Cottingham Road, Hull, HU6 7RX UK; 2 College of Veterinary Medicine, University of Wasit, Kut, Iraq; 3 Department of Medical Laboratories, College of Applied Medical Sciences, Majmaah University, Majmaah, Kingdom of Saudi Arabia; 4 Division of Cancer-Hull York Medical School, University of Hull, Cottingham Road, Hull, HU6 7RX UK

**Keywords:** Tissue factor, Apoptosis, Src-1, β1-Integrin

## Abstract

Accumulation of tissue factor (TF) within cells leads to cellular apoptosis mediated through p38 and p53 pathways. In this study, the involvement of Src1 in the induction of TF-mediated cell apoptosis, and the mechanisms of Src1 activation were investigated. Human coronary artery endothelial cell (HCAEC) were transfected with plasmids to express the wild-type TF (TF_Wt_-tGFP), or a mutant (Ser253 → Ala) which is incapable of being released from cells (TF_Ala253_-tGFP). The cells were then activated with PAR2-agonist peptide (SLIGKV-NH) and the phosphorylation of Src and Rac, and also the kinase activity of Src were assessed. Transfected cells were also pre-incubated with pp60c Src inhibitor, FAK inhibitor-14, or a blocking anti-β1-integrin antibody prior to activation and the phosphorylation of p38 as well as cellular apoptosis was examined. Finally, cells were co-transfected with the plasmids, together with a Src1-specific siRNA, activated as above and the cellular apoptosis measured. Activation of PAR2 lead to the phosphorylation of Src1 and Rac1 proteins at 60 min regardless of TF expression. Moreover, Src phosphorylation and kinase activity was prolonged up to 100 min in the presence of TF, with a significantly higher magnitude when the non-releasable TF_Ala253_-tGFP was expressed in HCAEC. Inhibition of Src with pp60c, or suppression of Src1 expression in cells, reduced p38 phosphorylation and prevented cellular apoptosis. In contrast, inhibition of FAK had no significant influence on Src kinase activity or cellular apoptosis. Finally, pre-incubation of cells with an inhibitory anti-β1-integrin antibody reduced both Src1 activation and cellular apoptosis. Our data show for the first time that the over-activation of Src1 is a mediator of TF-induced cellular apoptosis in endothelial cells through a mechanism that is dependent on its interaction with β1-integrin.

**Electronic supplementary material:**

The online version of this article (10.1007/s10495-019-01576-2) contains supplementary material, which is available to authorized users.

## Introduction

The accumulation of TF within the endothelial cells has been reported to contribute to the progression of chronic pathological disorders including cardiovascular disease [1–4] and cancer [4–7]. This occurs in addition to the procoagulant property of TF and is associated with the ability to regulate cellular processes such as migration and proliferation [8–14]. Furthermore, TF is capable of inducing cellular apoptosis through intracellular signalling mechanisms [15–17]. Recently, we reported that the accumulation of TF within endothelial cells, either through increased retention, or by acquisition of TF can promote cellular apoptosis [18, 19] through mechanisms mediated by p38 mitogen-activated protein kinases (MAPKs) [18]. TF-VIIa complex is capable of activating protease activated receptor (PAR)-2 which results in cellular signalling directly [11–14]. The formation of TF-fVIIa-PAR2 complex has also been shown to induce cellular signalling leading to gene expression and influences cellular proliferation, migration and angiogenesis [20, 21]. In addition, the interaction of TF with β1-integrin has recently been suggested to stimulate pro-angiogenic factors and to activate various signalling molecules including focal adhesion kinase (FAK) and p38 [10, 22–24].

p38 MAPK is a serine/threonine kinase that is activated by the phosphorylation of Thr180 and Tyr182 residues in response to different stress signals [25] and can promote cell apoptosis [26]. Upstream activators of p38 include Src1 [27, 28] and Rac1 [29, 30]. Src1 is also a component of the focal adhesion complex [31]. The activation of PAR2 has been shown to result in the phosphorylation of p38 [32]. Moreover, studies have reported that β1-integrin has the ability to induce FAK auto-phosphorylation at Tyr397, which in turn binds to the SH2 domain of Src1 protein [33, 34]. As a result of Src1-FAK complex formation, the intra-molecular interaction between the SH2 domain and the phospho-Tyr527 in the c-terminal of Src1 is disrupted [35]. The removal of the negative regulatory c-terminal creates an active conformation resulting in increased catalytic activity of Src1 [36] and occurs through the exposure of the activation loop which allows the phosphorylation of Src1 at Tyr416 [37, 38]. Rac1 also influences gene transcription through a number of signalling pathways including the c-Jun N-terminal kinase (JNK) and p38 MAPK pathway [39, 40]. Rac1 is activated by the phosphorylation of Ser71 [41]. In addition, previous studies have shown the cross-talk between Src1 and Rac1 [42, 43] resulting in indirect activation of downstream signalling pathways.

Both Src1 and Rac1 have been shown to be activated following the engagement of fVIIa and TF [44, 45]. Therefore, it is possible that Src1 and/or Rac1 may be capable of relaying the signal initiated by TF, and responsible for the activation of p38 leading to cellular apoptosis [18]. In this study, a previously used model for TF accumulation was used to identify and further elucidate the TF-induced apoptotic pathway. The model relies on the retention of TF, together with the activation of PAR2. The study aimed to elucidate the molecular mechanisms that mediate TF-signalling at the membrane level, resulting in the activation of p38.

## Materials and methods

### Cell culture, transfection and cellular activation

The pCMV6-Ac-TF-tGFP plasmid DNA (OriGene/Insight Biotechnology, Wembley, UK) was used to express wild-type or a variant form of TF, as previously described [46–48]. Preparations of pCMV6-Ac-tGFP (control) and pCMV6-Ac-TF_Ala253_-tGFP were described previously [46]. Human coronary artery endothelial cells (HCAEC), devoid of endogenous TF were cultured in MV media containing 5% (v/v) foetal calf serum (FCS) and growth supplements (PromoCell, Heidelberg, Germany). The initial experiments on Src phosphorylation time course were also carried out using human dermal blood microvascular cells (HDBEC) to ensure consistency between cells. Cells (2 × 10^5^) were seeded out into 12-well plates and transfected with 0.5 µg of pCMV6-Ac-TF_Wt_-tGFP, pCMV6-Ac-TF_Ala253_-tGFP or pCMV6-Ac-tGFP plasmid DNA. Transfection of the cells was carried out using TransIT-2020 (Geneflow, Lichfield, UK) according to the manufacturer’s instructions. Cells were permitted to express the proteins for 48 h and the expression of the TF variants was confirmed by flow cytometry [46, 49]. Prior to experiments, cells were pre-adapted to respective serum-free media for 1 h and then activated by incubation with PAR2-AP peptide (SLIGKV; 20 µM). In some experiments cells were pre-incubated with pp60c Src inhibitor (TSTEPQpYQPGENL; 0-500 µM) or a control peptide (TSTEPQWQPGENL) for 1 h, prior to activation with PAR2-AP. In other experiments the cells were pre-incubated for 2 h with FAK inhibitor-14 (1,2,4,5-benzenetetraamine tetrahydrochloride; 100 µM; Sigma Chemical Company Ltd, Poole, UK) or pre-incubated for 1 h with an inhibitory anti-β1-integrin antibody (AIIB2; 20 µg/ml; Developmental Studies Hybridoma Bank University of Iowa, Iowa City, USA) [21, 50] prior to activation. Finally, HCAEC were transfected with a specific set of SilencerSelect^®^-siRNA (10 pmol; Life Technologies, Paisley, UK) to suppress the expression of Src1 concurrently, or transfected with a comparable set of SilencerSelect^®^-negative control #1 siRNA (10 pmol; Life Technologies) for 48 h prior to activation. The concentration of siRNA to knock-down Src1 expression was optimised by western blot beforehand.

### Cell proliferation and apoptosis assays

Cell numbers were determined by staining with crystal violet as previously described [50, 51] and calculated from a standard curve. In addition, cellular apoptosis was quantified using the TiterTACS™ Colorimetric Apoptosis Detection Kit (AMS Biotechnology, Abingdon, UK) according to the manufacturer’s instructions and as described before [18, 19]. The criteria for endothelial cell apoptosis were evaluated according to the published consensus guidelines [52] and the apoptosis established as described previously [53].

### Analysis of Src, Rac, FAK and p38 phosphorylation by western blot

HCAEC (2 × 10^5^) were seeded out in 12-well plates and transfected to overexpress the wild-type or mutant form of TF-tGFP, or tGFP. In some experiments, the cells were co-transfected with Src1-siRNA or a control siRNA. Cells were activated with PAR2-AP (20 µM) and incubated for up to 2 h. The cells were then lysed in Laemmeli’s buffer containing a protease inhibitor cocktail (Sigma) and equal amounts were separated by 12% (w/v) SDS-PAGE. The protein bands were transferred onto nitrocellulose membranes and blocked with TBST (10 mM Tris–HCl pH 7.4, 150 mM NaCl, 0.05% Tween-20). To determine the total and phosphorylated Src1, the membranes were probed with a polyclonal rabbit anti-human Src1 antibody, diluted 1:3000 (v/v) in TBST and a rabbit monoclonal anti-human phospho-Tyr416 Src-family antibody (D49G4), diluted 1:4000 (v/v) in TBST (both from Cell Signalling Technologies/New England Biolabs, Hitchin, UK). Total and phospho-Rac1 were detected using a polyclonal rabbit anti-human Rac1/2/3 antibody diluted 1:3000 (v/v) in TBST, and a polyclonal rabbit anti-human phospho-Ser71-Rac antibody diluted 1:3000 (v/v) in TBST (Cell Signalling Technologies). Total and phosphorylated FAK were detected using a polyclonal rabbit anti-human FAK diluted 1:3000 (v/v) in TBST, and a monoclonal rabbit anti-human phospho-Tyr397-FAK (D20B1) diluted 1:3000 (v/v) in TBST, respectively (Cell Signalling Technologies). The membranes were then washed with TBST and probed with a goat anti-rabbit alkaline phosphatase-conjugated antibody (Santa Cruz Biotechnology, Heidelberg, Germany) diluted 1:1000 (v/v) and incubated for 90 min. The bands were then visualised using the Western Blue stabilised alkaline phosphatase-substrate (Promega) and recorded. Analysis of the phosphorylation of p38 was carried out as above but using a rabbit polyclonal anti-human p38 antibody diluted 1:3000 (v/v) in TBST and a monoclonal mouse anti-human phospho-Thr180/Tyr182-p38 MAPK antibody (28B10) diluted 1:3000 (v/v) in TBST (Cell Signalling Technologies) and then probed with a goat anti-rabbit alkaline phosphatase-conjugated antibody and a goat anti-mouse alkaline phosphatase-conjugated antibody (Santa Cruz Biotechnology) both diluted 1:2000 (v/v), respectively. The bands were then visualised using Western Blue substrate as above. All quantifications were normalised against GAPDH which was detected using a polyclonal goat anti-GAPDH antibody diluted 1:5000 (v/v) and then detected using an alkaline phosphatase-conjugated donkey anti-goat-IgG antibody diluted 1:2000 (v/v) obtained from Santa Cruz Biotechnology.

### Analysis of Src1 kinase activity

The ProFluor^®^ Src-family kinase assay (Promega Corporation, Southampton, UK) was used to quantify the Src1 enzymatic activity within the cells. The kit measures the tyrosine kinase activity of Src1 by its ability to catalyse the phosphorylation of a provided substrate molecule (Src-Family Kinase rhodamine 110; R110 substrate) the phosphorylation of which prevents further digestion with a provided protease. The amount of the released fluorescence substrate is therefore inversely proportional to the Src kinase activity and may be determined from a standard curve prepared using provided active Src. Briefly, HCAEC (2 × 10^5^/well) were seeded and transfected to express the wild-type TF-tGFP, the mutant form of TF (TF_Ala253_-tGFP), or tGFP as control, and incubated for 48 h to permit protein expression. Cells were then adapted to low-serum medium MV containing 2% (v/v) FCS for 60 min and activated with PAR2-AP (20 μM) for a further 90 min. The cells were then lysed in PhosphoSafe Extraction Reagent (Sigma) and the Src1 activity measured as follows. Cell lysates (5 μl) were placed in separate wells in opaque-walled 96-well plates and the kinase peptide substrate solution (20 μl) and ATP solution (25 μl) was added to each well and incubated with shaking for exactly 60 min at room temperature. The protease solution (25 μl) was then added and incubated for a further 60 min at room temperature and the reaction was then stopped using the provided stabiliser solution (25 μl). The fluorescence intensity of the released rhodamine was measured at Em 530 nm (Ex 485 nm), using the absorption of the included AMC at Em 460 nm (Ex 355 nm), as reference for the initial substrate concentration.

### Statistical analysis

All data represent the calculated mean values from the number of experiments stated in each figure legend ± the calculated standard error of the mean. Statistical analysis was carried out using the Statistical Package for the Social Sciences (SPSS Inc. Chicago, USA). Significance was determined using one-way ANOVA (analysis of variance) and Tukey’s honesty significance test or where appropriate, by paired *t* test.

## Results

### The presence of TF prolongs Src1 phosphorylation but not Rac1 phosphorylation following PAR2 activation

**Fig. 1:**
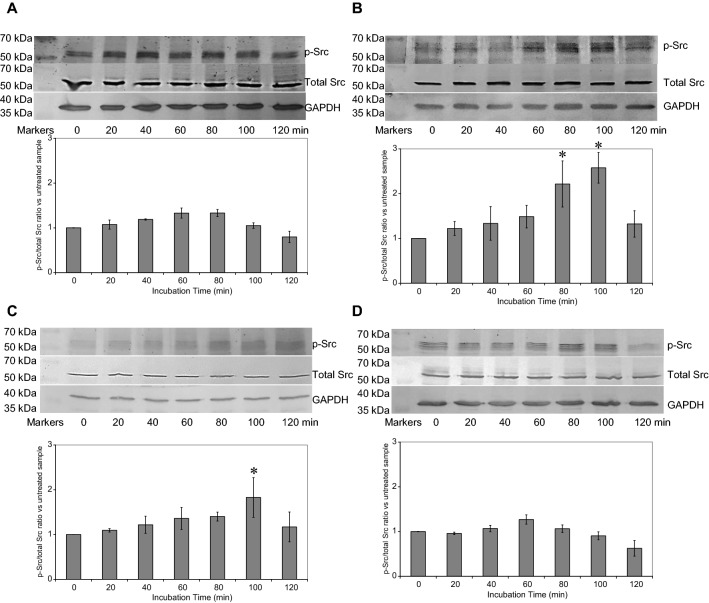
Analysis of Src phosphorylation by western blot. Human coronary artery endothelial cells (2 × 10^5^) were seeded out into 12-well plates and transfected with 0.5 µg of **a** pCMV6-Ac-tGFP, **b** pCMV6-Ac-TF_Ala253_-tGFP, **c** pCMV6-Ac-TF-tGFP plasmids, along with **d** an untransfected set of cells. The cells were incubated for 48 h to permit the expression of the recombinant proteins. Sets of cells were activated with PAR2-AP (20 µM) and incubated for up to 120 min. The cells were then lysed in Laemmeli’s buffer containing a protease inhibitor cocktail and separated by 12% (w/v) SDS-PAGE and transferred onto nitrocellulose membranes. The membranes were probed with a polyclonal rabbit anti-human Src1 antibody, diluted 1:3000 (v/v) or a rabbit monoclonal anti-human phospho-Tyr416-Src family antibody (D49G4), diluted 1:4000 (v/v) in TBST. The membranes were then washed and probed with a goat anti-rabbit alkaline phosphatase-conjugated antibody diluted 1:1000 (v/v) and then visualised using the Western Blue stabilised alkaline phosphatase-substrate and recorded. All quantifications were normalised against GAPDH which was detected using a polyclonal goat anti-GAPDH antibody diluted 1:5000 (v/v) and then detected using an alkaline phosphatase-conjugated donkey anti-goat-IgG antibody diluted 1:2000 (v/v). The ratios of phosphorylated to total Src were calculated using the ImageJ program. (n = 6; * = p < 0.05 vs. the untreated samples)

In the first instance, experiments were carried out to analyse the time-course of phosphorylation of Src and Rac and to determine the optimal time-point for measuring the activation of these two proteins. By expressing the TF in tandem with tGFP, it was possible to optimise the expression of the hybrid protein in the cells, prior to analysis. HCAEC were transfected to express TF_Wt_-tGFP, TF_Ala253_-tGFP, tGFP or use untransfected. All cells were then adapted to serum-free medium and activated by incubation with PAR2-AP (20 µM). Samples of cells were lysed at intervals up to 120 min and the phosphorylation of Src and Rac analysed by western blot. Activation of PAR2 in all cells resulted in a small rise in the phosphorylation of Src (Fig. 1) peaking at around 60 min. Moreover, on expression of either form of TF, the phosphorylation of Src was prolonged forming a second peak at around 100 min post-activation (Fig. 1b, c). This prolonging of the Src activation was most prominent on expression of the mutant form (TF_Ala253_-tGFP) but was observable with a lower magnitude on expression of the wild type (TF_Wt_-tGFP) in cells. To ensure consistency between various endothelial cell types as reported previously [52], the phosphorylation of Src was also examined at 0, 60 and 100 min in human dermal blood microvascular cells (HDBEC) which were transfected to express TF_Ala253_-tGFP (Supplementary Fig. 1). The activation of HDBEC also induced an increase in Src phosphorylation which was comparable to that obtained with HCAEC, and is consistent with previous studies regarding the responses of endothelial cells [54].

**Fig. 2:**
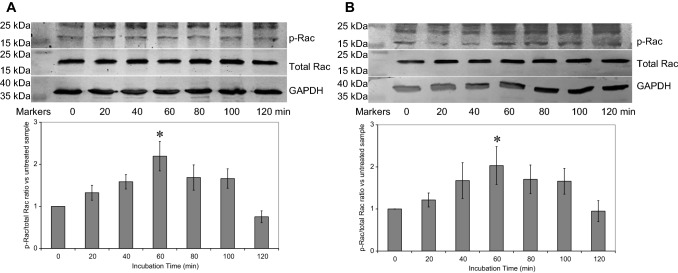
Analysis of Rac phosphorylation by western blot. Human coronary artery endothelial cells (2 × 10^5^) were seeded out into 12-well plates and transfected with 0.5 µg of **a** pCMV6-Ac-tGFP or **b** pCMV6-Ac-TF_Ala253_-tGFP (additional samples shown in Supplementary Fig. 2). The cells were incubated for 48 h to permit the expression of the recombinant proteins. Sets of cells were then activated with PAR2-AP (20 µM) and incubated for up to 120 min. The cells were then lysed and separated by 12% (w/v) SDS-PAGE and transferred onto nitrocellulose membranes. The membranes were probed with a polyclonal rabbit anti-human Rac1/2/3 antibody, diluted 1:3000 (v/v) and a rabbit monoclonal anti-human phospho-Ser71-Rac1 antibody, diluted 1:3000 (v/v) diluted in TBST. The membranes were then washed and probed with a goat anti-rabbit alkaline phosphatase-conjugated antibody diluted 1:1000 (v/v) and then visualised using the Western Blue stabilised alkaline phosphatase-substrate and recorded. All quantifications were normalised against GAPDH which was detected using a polyclonal goat anti-GAPDH antibody diluted 1:5000 (v/v) and then detected using an alkaline phosphatase-conjugated donkey anti-goat-IgG antibody diluted 1:2000 (v/v). The ratios of phosphorylated to total Rac were calculated using the ImageJ program. (n = 6; * = p < 0.05 vs. the untreated samples)

In contrast, activation of cells expressing tGFP (Fig. 2a), TF_Ala253_-tGFP (Fig. 2b), TF_Wt_-tGFP or the untransfected cells (Supplementary Fig. 2) produced a similar Rac-phosphorylation peak at around 60 min.

### The presence of TF amplifies Src1 phosphorylation and Src kinase activity following PAR2 activation

**Fig. 3:**
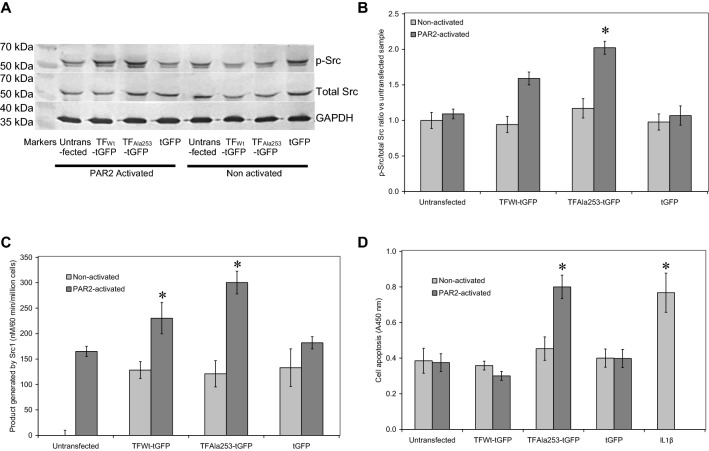
Analysis of Src phosphorylation, kinase activity and induction of cell apoptosis. Human coronary artery endothelial cells (2 × 10^5^) were seeded out into 12-well plates and transfected with 0.5 µg of pCMV6-Ac-tGFP, pCMV6-Ac-TF_Ala253_-tGFP, pCMV6-Ac-TF-tGFP plasmids, along with an untransfected set of cells. The cells were incubated for 48 h to permit the expression of the recombinant proteins. Sets of cells were then activated with PAR2-AP (20 µM) for 90 min. **a** The cells were then lysed and phosphorylated and total Src1 examined by western blot, as described before. **b** The ratios of phosphorylated to total Src were calculated using the ImageJ program. (n = 4; * = p < 0.05 vs. the untreated samples). **c** HCAEC (2 × 10^5^/well) were transfected as above and incubated for 48 h to permit protein expression. Cells were then adapted to low-serum medium MV containing 2% (v/v) FCS for 60 min and activated with PAR2-AP (20 μM) for a further 90 min. The cells were then lysed in PhosphoSafe Extraction Reagent and the Src activity measured using the ProFluor^®^ Src-family kinase assay. The fluorescence intensity of the released rhodamine was measured at Em 530 nm (Ex 485 nm), using the absorption of the included AMC at Em 460 nm (Ex 355 nm), as reference for the initial substrate concentration. (n = 5; * = p < 0.05 vs. the untreated samples). **d** HCAEC (2 × 10^5^/well) were transfected as above and incubated for 48 h to permit protein expression. Cells were then adapted to low-serum medium MV containing 2% (v/v) FCS for 60 min and activated with PAR2-AP (20 μM) for a further 90 min. The rate of cellular apoptosis was then quantified at 20 h, using the TiterTACS™ Colorimetric Apoptosis Detection Kit. (n = 4; * = p < 0.05 vs. the untreated samples)

Following establishing the optimal time-point for the measurement of Src phosphorylation, the magnitude of Src phosphorylation and the Src kinase activity in cells was compared in cells transfected with the wild-type and the mutant form of TF. Quantitative analysis of Src phosphorylation at 90 min post-activation with PAR2-AP (20 µM) confirmed the phosphorylation of Src at this time-point in the presence of wild-type TF and was significantly enhanced in the cells transfected to express TF_Ala253_-tGFP (Fig. 3a, b). Furthermore, analysis of the Src kinase activity at 90 min, mirrored the magnitudes of Src phosphorylation (Fig. 3c). Finally, quantification of the rate of cell apoptosis using a colourimetric TUNEL assay, indicated a putative association between Src activation and the induction of cell apoptosis (Fig. 3d).

### Src1 is a mediator of TF-induced p38 phosphorylation and cellular apoptosis

**Fig. 4:**
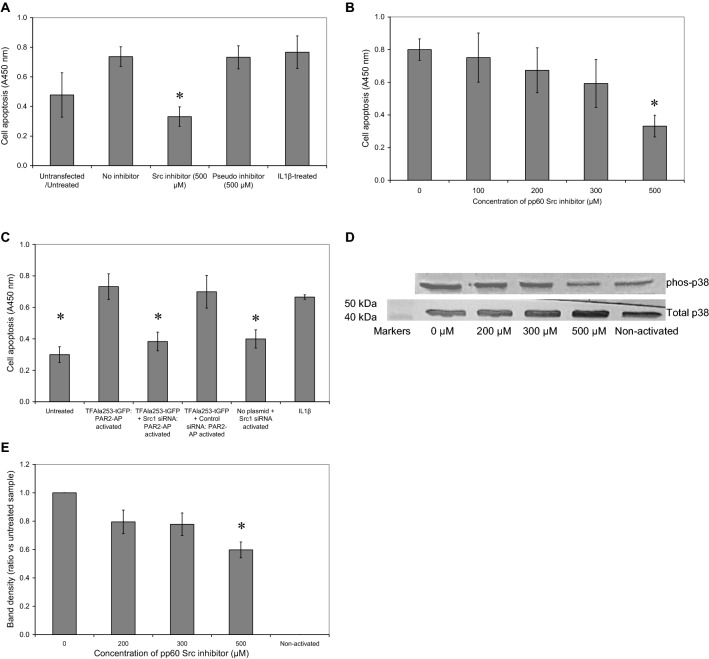
Examination of the involvement of Src1 in TF-mediated induction of cell apoptosis. Sets of HCAEC (2 × 10^5^/well) were transfected with pCMV6-Ac-TF_Ala253_-tGFP and incubated for 48 h to permit protein expression. **a** Cells were then adapted to low-serum medium MV containing 2% (v/v) FCS for 60 min together with the Src1 inhibitor pp60c Src inhibitor (TSTEPQpYQPGENL; 500 µM) and compared to a pseudo inhibitor (TSTEPQWQPGENL 500 nM). The cells were then activated with PAR2-AP (20 μM) for a further 90 min. A set of cells was also treated with IL-1β (10 ng/ml) as a positive control. The rate of cellular apoptosis was then quantified at 20 h, using the TiterTACS™ Colorimetric Apoptosis Detection Kit. (n = 4; * = p < 0.05 vs. sample devoid of inhibitor). **b** Sets of cells were transfected as above and then adapted to low-serum medium MV containing 2% (v/v) FCS for 60 min together with a range of Src1 inhibitor pp60c (0–500 nM). The cells were then activated with PAR2-AP (20 μM) for a further 90 min. The rate of cellular apoptosis was then quantified at 20 h, using the TiterTACS™ Colorimetric Apoptosis Detection Kit. (n = 4; * = p < 0.05 vs. the sample devoid of inhibitor). **c** Sets of HCAEC (2 × 10^5^/well) were co-transfected with pCMV6-Ac-TF_Ala253_-tGFP plasmid together with wither a Src1 siRNA, control siRNA or without any siRNA, and incubated for 48 h. The rate of cellular apoptosis was then quantified at 20 h, using the TiterTACS™ Colorimetric Apoptosis Detection Kit. (n = 4; * = p < 0.05 vs. sample devoid of siRNA). **d** HCAEC (2 × 10^5^) were transfected with pCMV6-Ac-TF_Ala253_-tGFP plasmid and incubated for 48 h to permit the expression of the recombinant protein. The cells were then activated with PAR2-AP (20 µM) and lysed and the amounts of phosphorylated-p38 and total-p38 were examined by western blot and **e** The ratios of phosphorylated to total p38 were calculated using the ImageJ program. (n = 4; * = p < 0.05 vs. sample devoid of inhibitor)

In order to examine the role of Src in TF induced apoptosis HCAEC (2 × 10^5^/well) were seeded in 12-well plates, incubated overnight and transfected with TFAla253-tGFP plasmid. Following 48 h of incubation, the cells were adapted to low-serum medium MV containing 2% (v/v) FCS for 60 min. The cells were pre-incubated for 60 min with pp60c Src1 inhibitor peptide (TSTEPQpYQPGENL) at a range of 0–500 μM, or alternatively with a pseudo-inhibitor peptide (TSTEPQWQPGENL; 500 μM) prior to activation with PAR2-AP (20 μM). Sets of cells were also treated with IL-1β (10 ng/ml) as a positive control and incubated for 24 h. Inhibition of Src in cells overexpressing TF_Ala253_-tGFP reduced the rate of cell apoptosis post-activation with PAR2-AP, in comparison to the samples treated with the pseudo-Src inhibitor or devoid of the inhibitor (Fig. 4a). This decrease in the rate of cell apoptosis on inclusion of the pp60c was dose dependent (Fig. 4b).

To further confirm the role of Src1 specifically, in TF-mediated apoptosis, HCAEC (2 × 10^5^/well) were co-transfected to express TF_Ala253_-tGFP together with Src1 siRNA or alternatively a control siRNA. The concentrations of the siRNA were optimised beforehand (Supplementary Fig. 3). The cells were adapted to low-serum medium MV containing 2% (v/v) FCS for 1 h prior to activation using PAR2-AP (20 μM). For comparison, sets of cells were treated with IL-1β (10 ng/ml) or used untreated. Cellular apoptosis was measured after 24 h post-activation using a colorimetric TUNEL assay. Suppression of Src expression reduced the rate of apoptosis to levels comparable to those of the untransfected/untreated cells (Fig. 4c) while co-transfection with the control siRNA did not exert any influence on TF-mediated cell apoptosis.

Previously, we showed that TF-mediated cell apoptosis involves the activation of p38 MAPK [18]. Therefore an attempt was made to decipher any association between TF, Src and p38 activation. HCAEC (2 × 10^5^/well) were transfected with TF_Ala253_-tGFP plasmid and incubated for 48 h. The cells were incubated with Src1 inhibitor (0–500 μM) for 60 min and then incubated for a further 90 min with PAR2-AP (20 μM). The cells were then lysed and analysed by western blot using mouse anti-human phospho-p38 and rabbit anti-human p38 antibodies. The ratios of phospho-p38: total p38 were then determined. In agreement with the above findings, incubation of cells with pp60c Src1 inhibitor peptide reduced p38 phosphorylation, following the activation of the cells (Fig. 4d, e).

### The activation of Src in the presence of TF is not dependent on FAK phosphorylation

**Fig. 5:**
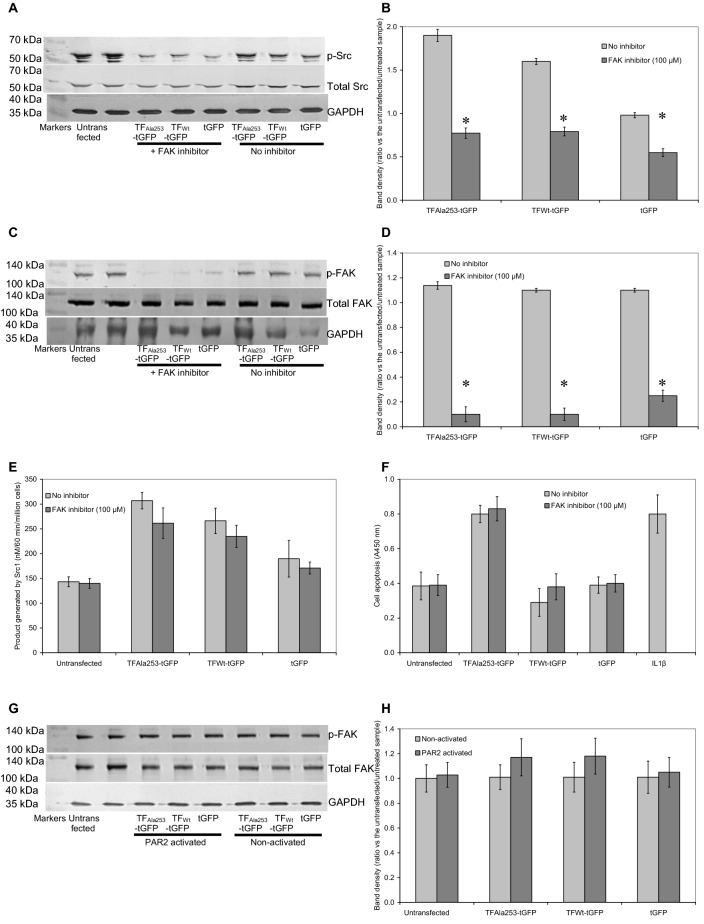
Examination of the involvement of FAK in TF-mediated induction of cell apoptosis. Sets of HCAEC (2 × 10^5^/well) were transfected with pCMV6-Ac-tGFP, pCMV6-Ac-TF_Ala253_-tGFP, pCMV6-Ac-TF-tGFP plasmids, along with an untransfected set of cells and incubated for 48 h to permit protein expression. Cells were then adapted to low-serum medium MV containing 2% (v/v) FCS for 60 min together with FAK inhibitor-14 (100 µM) and compared to vehicle control. The cells were then activated with PAR2-AP (20 μM) for a further 90 min. **a** The cells were then lysed and phosphorylated and total Src1 examined by western blot, as described before. **b** The ratios of phosphorylated to total Src were calculated using the ImageJ program. (n = 4; * = p < 0.05 vs. the untreated samples). **c** Phosphorylation of FAK was also analysed by western blot using specific antibodies to Tyr397-phosphorylated and total FAK. **d** The ratios of phosphorylated to total FAK were calculated using the ImageJ program. (n = 4; * = p < 0.05 vs. the untreated samples). **e** Sets of cells were treated as above and then lysed in PhosphoSafe Extraction Reagent and the Src kinase activity measured using the ProFluor^®^ Src-family kinase assay. (n = 4). **f** In addition, the rate of cellular apoptosis was then quantified at 20 h, using the TiterTACS™ Colorimetric Apoptosis Detection Kit. (n = 4). **g** Sets of HCAEC were transfected and adapted to serum-free medium as above. One set of cells were then activated with PAR2-AP (20 µM) and the level of FAK phosphorylation by western blot. **h** The ratios of phosphorylated to total FAK were calculated using the ImageJ program. (n = 4)

Recruitment of Src to focal adhesion clusters can result in the activation Src by FAK. Therefore, to assess the involvement of FAK in Src activation, HCAEC (2 × 10^5^/well) were cultured in 12-well plates and transfected to express TF_Wt_-tGFP, TF_Ala253_-tGFP, tGFP or were used untransfected and incubated for 48 h to express the proteins. The cells were pre-treated with the FAK inhibitor (FAK inhibitor-14; 100 µM) for 2 h. The duration of pre-incubation with 100 µM of the FAK inhibitor was optimised previously (Supplementary Fig. 4). The cells were then activated with PAR2-AP (20 μM) for 90 min and compared to non-activated cells by western blot. Analysis of phosphorylated and total Src antigens indicated a partial reduction in the phosphorylation of Tyr416 within Src, upon inclusion of FAK inhibitor-14 (Fig. 5a, b). Furthermore, analysis of the phosphorylation of Tyr397 within FAK, confirmed the significant decrease in the FAK activation (Fig. 5c, d). In contrast, measurement of Src kinase activity using the ProFluor^®^ Src-family kinase assay only showed a marginal reduction in the Src kinase activity following the inhibition of FAK (Fig. 5e). Importantly, the rate of cell apoptosis remained unaltered following the inhibition of FAK (Fig. 5f). To elucidate the mechanism further, the outcome of PAR2 activation on FAK phosphorylation, in cells expressing the various constructs was examined. Western blot analysis indicated that PAR2 activation had no influence on FAK phosphorylation which also remained unaltered by the expression of either form of TF-tGFP (Fig. 5g, h).

### Over-activation of Src1 by TF is dependent on β1-integrin

**Fig. 6:**
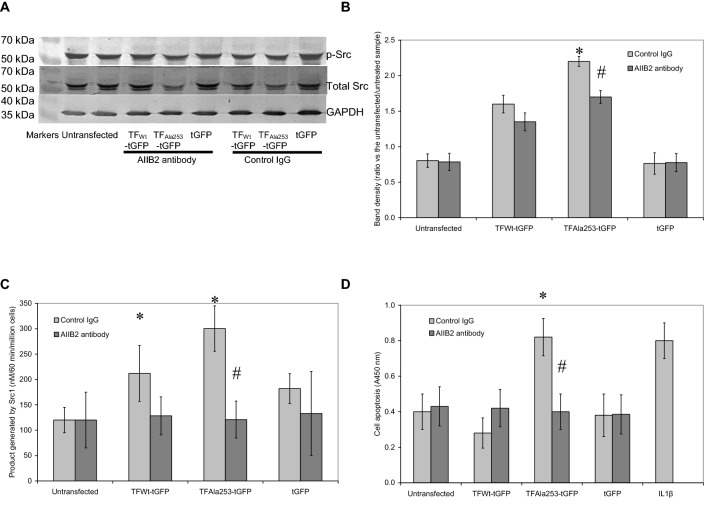
Examination of the involvement of β1-integrin in TF-mediated induction of cell apoptosis. Sets of HCAEC (2 × 10^5^/well) were transfected with pCMV6-Ac-tGFP, pCMV6-Ac-TF_Ala253_-tGFP, pCMV6-Ac-TF-tGFP plasmids, along with an untransfected set of cells and incubated for 48 h to permit protein expression. Cells were then adapted to low-serum medium MV containing 2% (v/v) FCS for 60 min together with a blocking anti-β1-integrin antibody (AIIB2; 20 µg/ml) and then were activated with PAR2-AP (20 μM) for a further 90 min. **a** The cells were then lysed and phosphorylated and total Src1 examined by western blot, as described before. **b** The ratios of phosphorylated to total Src were calculated using the ImageJ program. (n = 4; * = p < 0.05 vs. untreated samples; # = p < 0.05 vs. respective control IgG). **c** Sets of cells were treated as above and then lysed in PhosphoSafe Extraction Reagent and the Src kinase activity measured using the ProFluor^®^ Src-family kinase assay. (n = 4; * = p < 0.05 vs. the untreated samples; # = p < 0.05 vs. respective control IgG). **d** Sets of cells were treated as above and the rate of cellular apoptosis was quantified at 20 h, using the TiterTACS™ Colorimetric Apoptosis Detection Kit. (n = 4; * = p < 0.05 vs. the untreated samples; # = p < 0.05 vs. respective control IgG)

Finally, the role of β1-integrin in the activation of Src1, in cells overexpressing TF was evaluated. HCAEC (2 × 10^5^/well) expressing the mutant form of TF (TF_Ala253_-tGFP), wild-type TF_Wt_-tGFP, a control plasmid (tGFP) as well as an untransfected sample, were incubated with an inhibitory anti-β1-integrin antibody (AIIB2; 20 μg/ml) for 60 min [22, 50]. The cells were then activated using PAR2-AP (20 μM) for a further 90 min. Analyses of Src phosphorylation showed that blocking of β1-integrin marginally reduced the level of Src1 phosphorylation in cells expressing TF_Ala253_-tGFP (Fig. 6a, b). Moreover in agreement with the above, FAK phosphorylation remained unaffected (Supplementary Fig. 5). In contrast, incubation of cells with the inhibitory anti-β1-integrin antibody significantly reduced Src kinase activity (Fig. 6c). The change in Src kinase activity was also concurrent with substantial reduction in cellular apoptosis (Fig. 6d).

## Discussion

The release of TF during injury, trauma and infections has been well documented. High levels of circulating TF are also detected during chronic pathological conditions including cancer, diabetes and cardiovascular disease. Endothelial cells have been shown to be capable of acquiring exogenous TF, in vitro and in vivo [55, 56]. This accumulation of TF can overcome the cellular mechanisms of processing TF and as a result, the cells may become overloaded with TF. Abnormal levels of TF can induce cellular apoptosis which can give rise to endothelial dysfunction during disease conditions. Previously, we used a model for the accumulation of TF in endothelial cells to examine the mechanism of TF-induced cell apoptosis [15, 16]. Using this model we showed that the prevention of TF release through alanine-substitution of ser253 within its cytoplasmic domain, resulted in the induction of cell apoptosis through a mechanism involving p38 MAPK and p53. These in turn resulted in cell cycle arrest and the expression of bax [18]. In contrast, the expression of wild-type TF which is processed normally by the cells, lowered the rate of apoptosis and promoted cell proliferation. In this study, we have used this model to examine the signalling mediators in the activation of TF-induced apoptosis, but at the membrane-associated level. We have demonstrated the prolonged (> 90 min) phosphorylation of Src1 following the activation of PAR2, in cells expressing TF, but not in cells expressing the control plasmid, or untransfected cells. The phosphorylation of Src1 was concurrent with increased kinase activity of this protein. Importantly, accumulation of TF_Ala253_-tGFP further augmented this outcome, resulting in the over-activation of Src1. In contrast, the activation of Rac1 was unaltered by the presence of TF. This over-activation of Src1 was shown to be responsible for the high level of p38 phosphorylation and induction of cells apoptosis, since either inclusion of the Src inhibitor (pp60c) or siRNA mediated suppression of Src1 abolished the increased rate of cell apoptosis. The involvement of Src1 in the promotion of cell differentiation and proliferation has been thoroughly explored in the past [57, 58]. However, there are also some studies which show that the over-activation of Src can have a pro-apoptotic influence on cells [59]. Here, we have implicated Src1 as a mediator associating the disruptions in the cellular processing of TF, with the induction of apoptosis. These findings agree with the hypothesis that as a factor which appears early at the sight of injury, TF is ideally placed to have a dual function in instructing the cells to divide, or alternatively become apoptotic. Therefore, TF may act as a gauge for the level of injury and to distinguish between the severely injured cells and those which may be revived.

Previous studies have reported the ability of TF to induce cellular signalling through binding with the β1-integrin [22–24]. Furthermore, the interaction between FAK and Src1 is known to be capable of transmitting cellular signals initiated by β1-integrin [60, 61]. Therefore in the second part of the study, the possible roles of β1-integrin and FAK in TF-induced Src1 over-activation were examined. Chemical inhibition of FAK phosphorylation at Tyr397 using FAK inhibitor-14, reduced the phosphorylation of Tyr416 within Src1. This is in agreement with previous studies since Tyr397 phosphorylation within FAK is essential for the formation of Src1-FAK complex leading to the phosphorylation of Tyr416 in Src1. Moreover, it has been reported that the influence of β1-integrin on Src1 is mediated through activation of FAK-Src1 complex [62–64]. The clustering of integrins can promote the auto-phosphorylation of FAK at Tyr397 which generates a binding target for the SH2 domain within Src1 protein [33, 34]. The formation of this complex in turn disrupts the intra-molecular interaction between the SH2 domain and the regulatory phospho-Tyr527 in the c-terminal of Src1 [35]. The removal of this negative regulatory domain creates an active conformation through the exposure of the activation loop. This in turn results in the phosphorylation of Src1 at Tyr416 [37, 38] and increased catalytic activity of Src1 [36]. However in our study, only a marginal reduction in Src1 kinase activity was detected following the inhibition of FAK in cells expressing the mutant or wild-type TF. It has recently been suggested that FAK inhibition is not sufficient to completely suppress Src1 activity since the lack of Tyr397 phosphorylation in FAK alone, is not sufficient for preventing Src1 activation [65]. Consequently, Src1 may exist in a state where both Tyr416 and Tyr527 remain phosphorylated within this protein [66] and may be activated by mechanisms that are independent of FAK [65–68]. Additionally, previous studies have reported that the α4β1-integrin is capable of activating Src1 independently of FAK [69] through mechanism involving other members of the focal adhesion complex such as paxillin and vinculin, which were reported as binding proteins of Src1 at SH3 [70]. It has also been shown that the over-expression of v-Src in FAK-deficient cell lines results in the formation of avascular tumours in mice suggesting that Src activity alone, may in fact prevent vascularisation [71]. Therefore, FAK-Src signalling appears to be essential for the paracrine signalling between tumour cells and endothelial cells, which is mediated through the induction of VEGF expression in the latter cells [72]. However in our study, Src appeared to be activated without FAK activation. Therefore, the over-activation of Src alone, in endothelial cells, in response to TF may be detrimental to vascular proliferation.

Blocking of β1-integrin using an inhibitory antibody (AIIB2), in cells expressing either form of TF, reduced both the phosphorylation of Src1 and the associated kinase activity, following PAR2 activation. Further analysis of FAK phosphorylation using western blot, in cells pre-incubated with β1-integrin blocking antibody did not show any reduction in FAK phosphorylation (Supplementary Fig. 5). However, this antibody (AIIB2) has been shown to reduce FAK phosphorylation at Tyr397 [67], and the underlying mechanism is thought to involve the interference with integrin clustering. This clustering is also essential for the maximal activation of Src1 [68, 69]. Therefore, in agreement with the above, Src1 appears to be activated by mechanisms that are independent of FAK [65]. As mentioned above, other studies have also demonstrated that the α4β1-integrin is capable of activating Src1 independently of FAK [70], and through mechanisms involving other members of the focal adhesion complex. In these mechanisms, the binding Src1 occurs through SH3 domain [71]. Therefore, the observed decrease in Src1 phosphorylation, following the blocking of β1-integrin using AIIB2 antibody, appears to involve reduced recruitment of Src1 protein to the focal adhesion complex, through preventing the interaction with other focal adhesion components. Finally, a higher level of Src1 activity was detected in transfected cells than the untransfected control cells and was highest in cells expressing the mutant TF_Ala253_-tGFP protein. This further indicates a possible direct interaction between the expressed TF and β1-integrin, even in the absence of PAR2 activation.

In conclusion, this study has identified a crucial role for Src1 over-activation, in mediating TF signalling, resulting in the activation of p38 and subsequent apoptosis in HCAEC. In addition, for the first time β1-integrin has been shown to act as signalling intermediary associating increased cellular levels of TF with the over-activation of Src1. Therefore, this study has identified a crucial step in the mechanism of TF-induced apoptosis. The activation of this pathway determines the fate of the cells that come into contact with TF, and may be crucial during inflammatory conditions such as cancer and vascular disease.

## Electronic supplementary material

Below is the link to the electronic supplementary material.

Supplementary material 1 (DOC 2141 kb)
