# Supplementary material for: Accumulation of tissue factor in endothelial cells promotes cellular apoptosis through over-activation of Src1 and involves β1-integrin signalling
Source: Apoptosis. 2019 Oct 25;25(1):29–41. doi: 10.1007/s10495-019-01576-2 (PMC6965344; doi:10.1007/s10495-019-01576-2)
Supplement: Supplementary file 1 — Supplementary material 1 (DOC 2141 kb) [file 10495_2019_1576_MOESM1_ESM.doc]

Supplementary figure 1

A)


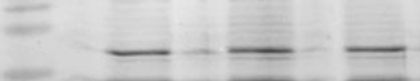


Markers 0 60 100 min

70 kDa

50 kDa

70 kDa

50 kDa

40 kDa

35 kDa

p-Src

Total Src

GAPDH

B)

*

Analysis of Src phosphorylation IN HDBEC by western blot. Human dermal blood endothelial cells (2 × 10^5^) were seeded out into 12-well plates and transfected with 0.5 µg of pCMV6-Ac-TF_Ala253_-tGFP. The cells were incubated for 48 h to permit the expression of the recombinant proteins. Sets of cells were activated with PAR2-AP (20 µM) and incubated for 0-100 min. The cells were then lysed in Laemmeli’s buffer containing a protease inhibitor cocktail and separated by 12% (w/v) SDS-PAGE and transferred onto nitrocellulose membranes. The membranes were probed with a polyclonal rabbit anti-human Src1 antibody, diluted 1:3000 (v/v) or a rabbit monoclonal anti-human phospho-Tyr416-Src family antibody (D49G4), diluted 1:4000 (v/v) in TBST. The membranes were then washed and probed with a goat anti-rabbit alkaline phosphatase-conjugated antibody diluted 1:1000 (v/v) and then visualised using the Western Blue stabilised alkaline phosphatase-substrate and recorded. All quantifications were normalised against GAPDH which was detected using a polyclonal goat anti-GAPDH antibody diluted 1:5000 (v/v) and then detected using an alkaline phosphatase-conjugated donkey anti-goat-IgG antibody diluted 1:2000 (v/v). The ratios of phosphorylated to total Src were calculated using the ImageJ program. (n = 3; * = p< 0.05 vs the untreated samples).

Supplementary figure 2

A) B)

25 kDa

15 kDa

25 kDa

15 kDa

40 kDa

35 kDa

25 kDa

15 kDa

25 kDa

15 kDa

40 kDa

35 kDa

p-Rac

Total Rac

GAPDH

p-Rac

Total Rac

GAPDH

Markers 0 20 40 60 80 100 120 min Markers 0 20 40 60 80 100 120 min

Additional analysis of Rac phosphorylation by western blot. Human coronary artery endothelial cells (2 × 10^5^) were seeded out into 48-well plates and transfected with 0.5 µg of A) pCMV6-Ac-TF-tGFP plasmids, along with B) an untransfected set of cells. The cells were incubated for 48 h to permit the expression of the recombinant proteins. Sets if cells were then activated with PAR2-AP (20 µM) and incubated for up to 120 min. The cells were then lysed in Laemmeli’s buffer containing a protease inhibitor cocktail and separated by 12% (w/v) SDS-PAGE and transferred onto nitrocellulose membranes. The membranes were probed with a polyclonal rabbit anti-human Rac1/2/3 antibody, diluted 1:3000 (v/v) and a rabbit monoclonal anti-human phospho-Ser71-Rac1 antibody, diluted 1:3000 (v/v) diluted in TBST. The membranes were then washed and probed with a goat anti-rabbit alkaline phosphatase-conjugated antibody diluted 1:1000 (v/v) and then visualised using the Western Blue stabilised alkaline phosphatase-substrate and recorded. All quantifications were normalised against GAPDH which was detected using a polyclonal goat anti-GAPDH antibody diluted 1:5000 (v/v) and then detected using an alkaline phosphatase-conjugated donkey anti-goat-IgG antibody diluted 1:2000 (v/v). The ratio of phosphorylated to total Rac was calculated using the ImageJ program. (n = 6).

Supplementary figure 3

70 kDa

50 kDa

Src1


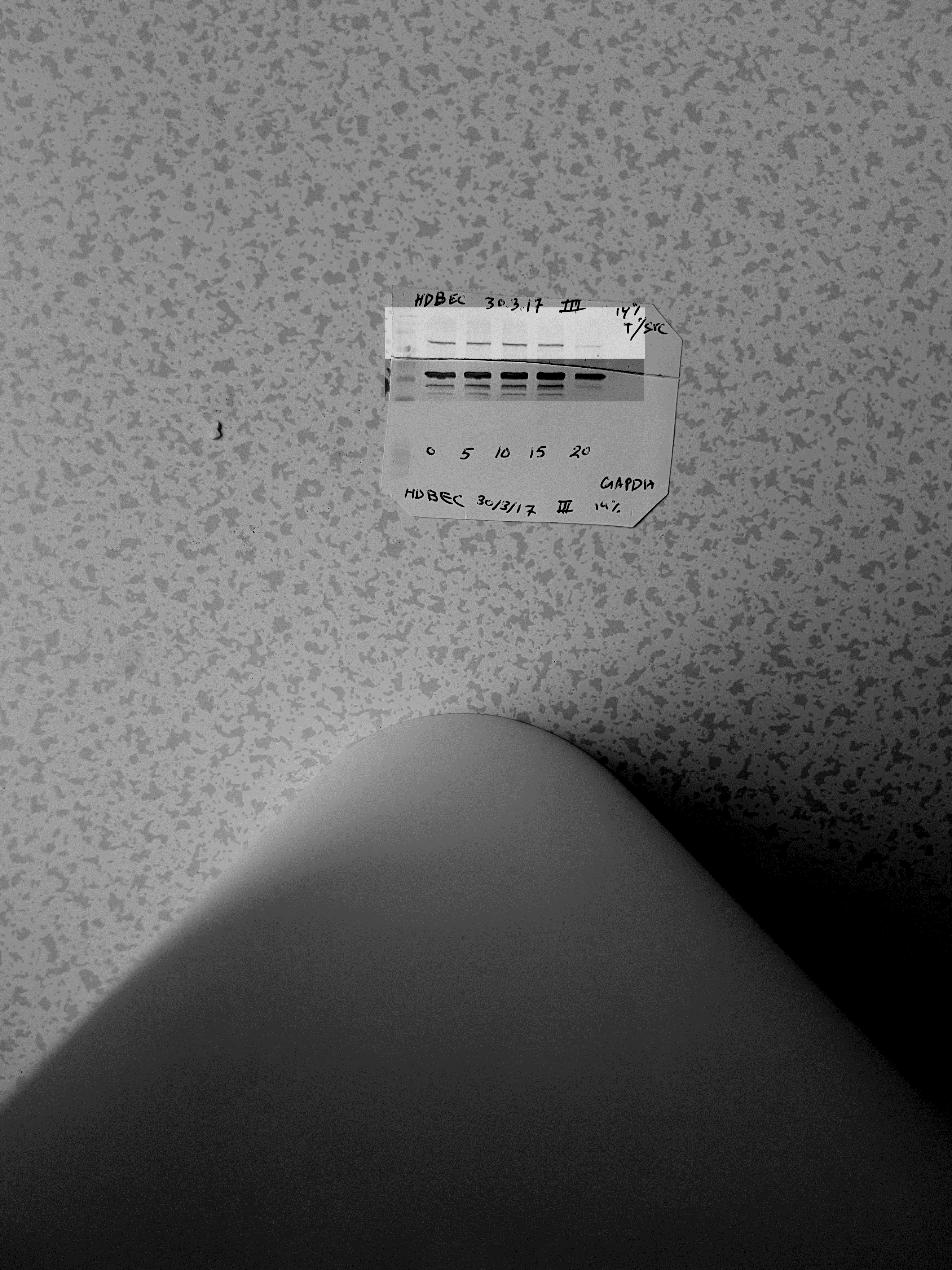


Markers 0 50 100 150 200 nM of Src1-siRNA

Optimisation of Src1 knock down using siRNA. Sets of HCAEC (2 × 10^5^/well) were transfected with a range of concentrations of a specific Src1 siRNA and the incubated for 48 h. The cells were then lysed in Laemmeli’s buffer containing a protease inhibitor cocktail and separated by 12% (w/v) SDS-PAGE and transferred onto nitrocellulose membranes. The membranes were probed with a polyclonal rabbit anti-human Src1 antibody, diluted 1:3000 (v/v) in TBST. The membranes were then washed and probed with a goat anti-rabbit alkaline phosphatase-conjugated antibody diluted 1:1000 (v/v) and then visualised using the Western Blue stabilised alkaline phosphatase-substrate and recorded. (n = 3).

Supplementary figure 4

140 kDa

100 kDa

140 kDa

100 kDa

35 kDa

p-FAK

Total FAK

GAPDH

Markers 0 1 2 3 4 5 Incubation time (h)

Optimisation of FAK inhibition. Sets of HCAEC (2 × 10^5^/well) were incubated with the inhibitor "FAK inhibitor-14" (100 µM) for up to 5 h. The cells were then lysed in Laemmeli’s buffer containing a protease inhibitor cocktail and phosphorylation of FAK was analysed by western blot using specific antibodies to Tyr397-phosphorylated and total FAK. All measurements were normalised against GAPDH (n = 3).

Supplementary figure 5

140 kDa

100 kDa

140 kDa

100 kDa

40 kDa

35 kDa

p-FAK

Total FAK

GAPDH

Markers Untrans TF_Wt_ TF_Ala253_ tGFP Untrans TF_Wt_ TF_Ala253_ tGFP

-fected -tGFP -tGFP -fected -tGFP -tGFP

With AIIB2 Without AIIB2

Analysis of the influence of β1-integrin inhibition on FAK phosphorylation. Sets of HCAEC (2 × 10^5^/well) were transfected with pCMV6-Ac-tGFP, pCMV6-Ac-TF_Ala253_-tGFP, pCMV6-Ac-TF-tGFP plasmids, along with an untransfected set of cells and incubated for 48 h to permit protein expression. Cells were then adapted to low-serum medium MV containing 2 % (v/v) FCS for 60 min together with a clocking anti-β1-integrin antibody (AIIB2; 20 µg/ml). The cells were then activated with PAR2-AP (20 μM) for a further 90 min. The cells were then lysed in Laemmeli’s buffer containing a protease inhibitor cocktail and phosphorylated and the phosphorylation of FAK was analysed by western blot using specific antibodies to Tyr397-phosphorylated and total FAK. (n = 4).
